# Secondary sexual ornamentation and non-additive genetic benefits of female mate choice

**DOI:** 10.1098/rspb.2007.0063

**Published:** 2007-03-20

**Authors:** Jane M Reid

**Affiliations:** School of Biological Sciences, Zoology Building, University of AberdeenTillydrone Avenue, Aberdeen AB24 2TZ, UK

**Keywords:** heterozygosity, inbreeding depression, indirect fitness benefits, intersexual selection, relatedness

## Abstract

Ornamental secondary sexual traits are hypothesized to evolve in response to directional mating preferences for more ornamented mates. Such mating preferences may themselves evolve partly because ornamentation indicates an individual's additive genetic quality (good genes). While mate choice can also confer non-additive genetic benefits (compatible genes), the identity of the most ‘compatible’ mate is assumed to depend on the choosy individual's own genotype. It is therefore unclear how choice for non-additive genetic benefits could contribute to directional mating preferences and consequently the evolution of ornamentation. In free-living song sparrows (*Melospiza melodia*), individual males varied in their kinship with the female population. Furthermore, a male's song repertoire size, a secondary sexual trait, was negatively correlated with kinship such that males with larger repertoires were less closely related to the female population. After excluding close relatives as potential mates, individual females were on average less closely related to males with larger repertoires. Therefore, female song sparrows expressing directional preferences for males with larger repertoires would on average acquire relatively unrelated mates and produce relatively outbred offspring. Such non-additive genetic fitness benefits of directional mating preferences, which may reflect genetic dominance variance expressed in structured populations, should be incorporated into genetic models of sexual selection.

## 1. Introduction

The precise mechanisms driving the evolution of elaborate ornamental secondary sexual traits constitute an enduring puzzle in evolutionary ecology (Andersson 1994; Andersson & Simmons 2006). At one level, the evolution of ornamentation can be explained as the outcome of sexual selection, imposed by directional mating preferences for more ornamented mates (Darwin 1871; Andersson 1994). Such directional mating preferences are widely observed, most frequently concerning female choice for more ornamented males (Andersson 1994; Kokko *et al*. 2003). However, the evolutionary mechanisms that cause and maintain such mating preferences, and therefore drive the evolution of ornamentation, remain contentious, particularly when direct fitness benefits of mate choice are minimal (Kokko *et al*. 2003). Characterizing the nature and magnitude of indirect genetic benefits of female choice for more ornamented males therefore remains a major goal in evolutionary ecology, and is integral to understanding the evolution and maintenance of intersexual selection (Møller & Alatalo 1999; Tomkins *et al*. 2004; Neff & Pitcher 2005; Andersson & Simmons 2006; Kokko *et al*. 2006; Qvarnström *et al*. 2006).

One major hypothesis is that females gain additive genetic fitness benefits by expressing directional mating preferences for more ornamented males, reflecting the direct inheritance of beneficial alleles by offspring from more ornamented fathers (choice for additive ‘good genes’; Andersson 1994; Neff & Pitcher 2005; Kokko *et al*. 2006). This hypothesis has prompted considerable research and there is now evidence that ornamentation can indicate components of a male's additive genetic quality and therefore that directional female preferences may reflect selection for indirect benefits in the form of heritable components of fitness (Møller & Alatalo 1999; Neff & Pitcher 2005; Qvarnström *et al*. 2006, although see Kokko *et al*. 2003; Hunt *et al*. 2004). However, despite such empirical support, it remains unclear how female choice for additive genetic benefits is maintained, given that any unanimous directional female preference is expected to deplete additive genetic variance for fitness and consequently eliminate the benefit of choice (Kirkpatrick & Ryan 1991; Rowe & Houle 1996; Kokko *et al*. 2003). Several possible resolutions of this paradox have been proposed, but debates continue (Hamilton & Zuk 1982; Rowe & Houle 1996; Tomkins *et al*. 2004; Kokko *et al*. 2006).

In parallel, recent studies increasingly emphasize the role of non-additive genetic benefits in driving female mate choice. Evidence is accumulating that females may preferentially mate with genetically compatible, dissimilar or less closely related males, thereby producing relatively heterozygous, genetically diverse or outbred offspring (choice for broadly defined ‘compatible genes’; Widemo & Sæther 1999; Tregenza & Wedell 2000; Mays & Hill 2004; Neff & Pitcher 2005). Since fitness frequently declines with inbreeding and with reduced heterozygosity and genetic diversity (Hansson & Westerberg 2002; Keller & Waller 2002), such mating preferences are likely to increase offspring fitness. Female choice for non-additive genetic benefits may not deplete genetic variance and therefore be evolutionarily robust (Reinhold 2002; Lehmann *et al*. 2006). However, models of female choice for non-additive benefits are generally accepted to predict that the identity of each female's optimal mate will depend on the female's own genotype. Females are therefore predicted to show individual (idiosyncratic) rather than unanimous directional mating preferences (Neff & Pitcher 2005). Such individually ‘compatible’ mates may be identified by pheromone matching or other means of specific direct comparison, rather than by assessing ornamentation (Widemo & Sæther 1999; Tregenza & Wedell 2000; Colegrave *et al*. 2002). Consequently, it is not clear how female choice for non-additive genetic benefits might contribute to the evolution of directional female preferences for more ornamented males and therefore the evolution of ornamentation. A demonstration that a directional female preference for more ornamented males could in fact confer a general non-additive genetic benefit of mate choice would therefore suggest an extra dimension to existing genetic models of the causes, consequences and maintenance of intersexual selection (see Colegrave *et al*. 2002; Mays & Hill 2004; Neff & Pitcher 2005).

The apparent dichotomy between directional female preferences for more ornamented males with additive good genes and individual female choice for males with non-additive compatible genes (Colegrave *et al*. 2002; Mays & Hill 2004) could be partially reconciled if the ornament subject to female choice were to indicate some component of a male's general genetic dissimilarity from the female population. In this case, a directional female preference for more ornamented males could translate into general choice for a relatively dissimilar male, and a consequent non-additive genetic fitness benefit in terms of the production of relatively outbred and/or genetically diverse offspring. This mechanism requires that individual males vary in their relatedness to or dissimilarity from the female population, and that relatedness and/or dissimilarity is correlated with the expression of a secondary sexual trait subject to a directional female preference.

Here, I consider these conditions with reference to a free-living population of song sparrows (*Melospiza melodia*) on Mandarte Island, Canada, for which substantial pedigree data exist. I first show that a male's mean kinship with the female population varied substantially among males. Second, I show that a male's song repertoire size, a secondary sexual trait, was correlated with its mean kinship such that males with larger song repertoires were less closely related to the female population. Finally, I investigate whether male repertoire size was correlated with kinship within individual females, and therefore whether a directional preference for males with larger repertoires could translate into a non-additive genetic benefit of mate choice (manifested as the production of relatively outbred offspring) for individual females. I consider the mechanisms underlying observed correlations between ornamentation and kinship, and discuss their possible generality and implications for genetic models of intersexual selection.

## 2. Material and methods

### (a) Study population

Mandarte Island, approximately 6 ha in size, lies 25 km northeast of Victoria, British Columbia, Canada. Its small (35±3 pairs on average) resident population of song sparrows (*M. melodia*) has been studied intensively since 1975 (Smith *et al*. 2006). Throughout this long-term study, all song sparrows fledged on Mandarte have been individually colour ringed before leaving their natal territory or shortly thereafter. All immigrants to the breeding population have been individually colour ringed soon after arrival. All population members are therefore individually identifiable. In each year, all surviving population members have been identified, and all social pairings and breeding attempts have been monitored and documented (Smith *et al*. 2006). On Mandarte, song sparrows typically breed up to three times during March–July starting from their first summer (although some males remain unmated for whole or part seasons). Recruits survive 2.3 seasons on an average (range 1–9 seasons; Smith *et al*. 2006).

Based on these detailed long-term data, a complete social pedigree has been compiled for the population, covering all sparrows fledged since 1981 (Keller 1998). Standard pedigree algorithms can therefore be used to estimate each individual's coefficient of inbreeding (*f*) and the coefficient of kinship (*k*) between any male–female pairing (Falconer & Mackay 1996; Keller 1998; Reid *et al*. 2006). The coefficient of inbreeding, *f*, reflects the probability that a pair of homologous alleles will be identical by descent; a high *f* indicates a relatively inbred and therefore relatively homozygous individual. The coefficient of kinship, *k*, measures the relatedness between a male–female pair and equals the *f* of offspring that would be produced by that pairing; a high *k* indicates a closely related pair whose offspring would be relatively inbred. Substantial and repeatable inbreeding depression in survival, reproduction, immune response and song repertoire size has been observed in song sparrows on Mandarte (Keller 1998; Reid *et al*. 2003, 2005, 2007; Smith *et al*. 2006).

Although song sparrows are primarily socially monogamous, microsatellite genotyping showed that approximately 25% of offspring hatched on Mandarte during 1993–1996 had extra-pair sires, while none mismatched their social mother (O'Connor *et al*. 2006). Given similar extra-pair paternity rates in other years, approximately 13% of links within the social pedigree will be incorrect. However, during 1993–1996, extra-pair paternities were not more frequent in females that were more closely related to their social mate, and females did not choose extra-pair sires that were more or less closely related than their social mate (see Reid *et al*. 2007). Furthermore, genetic and social estimates of reproductive skew did not differ significantly (O'Connor *et al*. 2006). Extra-pair paternities are therefore expected to introduce error but not bias into the estimates of *f* and *k*.

### (b) Song repertoire size

Male song sparrows sing complex songs consisting of repertoires of 4–13 distinct song types (Searcy & Marler 1981; Beecher *et al*. 2000). Males learn their songs during their first autumn (not necessarily from their fathers or natal neighbours), and then retain the same repertoire for life (Cassidy 1993; Beecher *et al*. 2000). In captivity, oestradiol-treated female song sparrows performed more copulation solicitation displays in response to artificial playback of larger song repertoires (Searcy & Marler 1981). Furthermore, on Mandarte, males with larger repertoires were more likely to mate and bred earlier during their first year (Reid *et al*. 2004). Experimental and correlative evidence is therefore consistent with the hypothesis that female song sparrows preferentially mate with males with larger song repertoires, and therefore that song repertoire size is a secondary sexual trait subject to female choice (see Searcy 1984; Beecher *et al*. 2000; Reid *et al*. 2004 for discussion).

In 2003, I recorded the full song repertoire sizes of 22 out of 31 adult male song sparrows alive on Mandarte. All 22 recorded males were hatched on Mandarte (rather than immigrants). Two unrecorded males were non-territorial floaters that did not sing or breed. The remaining seven unrecorded males had retained mates and territories from previous years and rarely sang. The 22 recorded males therefore probably comprised the full set of males available as primary mates in 2003. Songs were recorded using an Optimus CTR-117 recorder and Sennheiser ME67 microphone and analysed using Syrinx (John Burt, www.syrinxpc.com). A mean of 350±10 (range 225–465) continuously recorded songs, including at least 20 distinct song blocks, were typed for each male. Since 225 songs are sufficient to estimate repertoire size with 99% confidence on Mandarte (Cassidy 1993), each male's full song repertoire size was measured with high confidence (Reid *et al*. 2004).

### (c) Analyses

Analyses focused on the adult song sparrow population alive on Mandarte in 2003 (the year in which songs were recorded), which comprised *n*_m_=31 males and *n*_f_=26 females. I first used the population pedigree to estimate the coefficient of kinship (*k*) between every possible male–female pair that could have formed within the population (giving *n*_f_ values of *k* for each of the *n*_m_ males). I quantified each male's mean kinship (*k*_m_) with the female population (where *k*_m_=*∑k*/*n*_f_), and tested whether *k*_m_ was correlated with song repertoire size across males. Since within-male distributions of *k* were right skewed in some cases, I repeated the analyses using median kinship (*k*_med_). However, since analyses based on *k*_m_ and *k*_med_ gave qualitatively identical results and *k*_m_ may better predict the long-term evolutionary consequence of selection, I solely present results based on *k*_m_.

A correlation between song repertoire size and *k*_m_ would imply that a male's repertoire size indicates its mean kinship with the female population. However, since population-level correlations do not necessarily reflect effects operating in individuals, such a correlation would not necessarily imply that repertoire size reliably predicts variation in *k* between any individual female and the set of available males. Therefore, to assess the value of repertoire size as an indicator of the kinship between an individual female and any specific male, I used general linear models to test whether male repertoire size was correlated with *k* within individual females.

Since my main aim was to describe overall correlations between kinship and song repertoire size arising in a natural population (and thereby investigate the genetic benefits of directional mating preferences that could potentially arise), I initially considered all opposite-sex population members as potential mates of each focal individual. However, analyses were greatly influenced by the inclusion of close relatives of each focal individual (parents, full and half sibs, offspring and grand-offspring) in the set of potential mates, which formed outliers with respect to *k* (§3). Since animals are widely suggested to recognize or otherwise avoid mating with close kin (Pusey & Wolf 1996; Komdeur & Hatchwell 1999; Mateo & Johnston 2000), I repeated the analyses after excluding close relatives (*k*≥0.125; i.e. half-sibs, grand-offspring, grandparents and closer relatives) from each individual's set of potential mates.

Since estimates of *k* between each focal individual and multiple opposite-sex population members are not independent and distributions of *k* were right skewed in some cases, probabilities associated with observed effect sizes were estimated using randomization procedures (Manly 2007). Dependent variables were randomized and observed test statistics was compared with the distribution generated over 10 000 iterations. However, I place primary emphasis on the estimated effect sizes. Analyses were run in R (v. 2.4.0) and Pedigree Viewer (http://www-personal.une.edu.au/∼bkinghor/pedigree.htm). Means±1 s.e. are presented.

## 3. Results

### (a) Variation in kinship

Across all 31 male song sparrows alive on Mandarte in 2003, estimated *k*_m_ varied from 0.013 to 0.108 (mean 0.077±0.004) including all 26 females as potential mates of each male, and from 0.009 to 0.076 (mean 0.057±0.003) after excluding close female relatives of each male (*n*=22.3±0.4 females for each male). Estimated *k*_m_ therefore varied up to eightfold among males.

### (b) Song repertoire size and mean kinship

**Figure 1:**
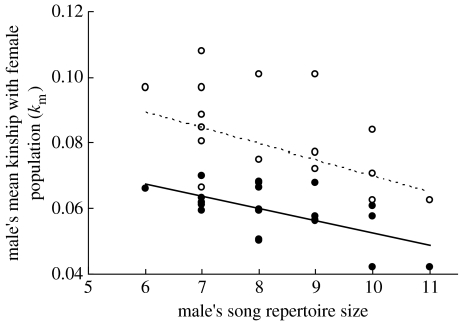
Relationships between a male song sparrow's song repertoire size and mean kinship (*k*_m_) with the female population. Males with larger song repertoires were less closely related to the female population, including all females in the set of potential mates (open symbols, dashed line, *n*=22, *r*=−0.43, *p*=0.045, *R*^2^=0.18) and excluding close female relatives of each focal male (filled symbols, solid line, *n*=22, *r*=−0.54, *p*=0.010, *R*^2^=0.32). Regression lines are shown for clarity.

Across 22 males whose songs were recorded, song repertoire size averaged 8.1±0.3 (range 6–11, CV=0.16). Repertoire size was negatively correlated with *k*_m_ both including all 26 females as potential mates of each male and excluding close female relatives of each male (figure 1). On average, male song sparrows with larger song repertoires were less closely related to the female population.

### (c) Song repertoire size and individual kinship

**Figure 2:**
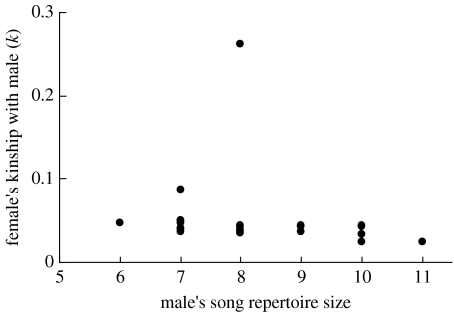
Relationship between male song repertoire size and kinship (*k*) for one example female. This female had one close male relative (a brother) on Mandarte in 2003.

**Figure 3:**
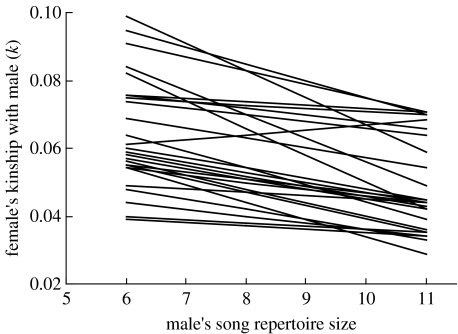
Relationships between male song repertoire size and kinship (*k*) within each of 26 female song sparrows (after excluding close relatives, *k*≥0.125, of each female as potential mates). Overall, *k* declined with increasing repertoire size within individual females (repertoire size *F*_1488_=32.4, *p*<0.001; female *F*_25 488_=11.4, *p*<0.001; female×repertoire size *F*_25 488_=0.7, *p*=0.97, *n*=18.8±0.5 males for each female). Regression lines are shown for each individual female. For clarity, intercepts have been adjusted where relationships were identical for multiple females.

Including all 22 males with known repertoire sizes as potential mates of each female, *k* was on average negatively correlated with song repertoire size within individual females (repertoire size *F*_1571_=6.3, *p*=0.005; female *F*_25 571_=3.1, *p*=0.001). However, estimated effect sizes varied among females and were small on average (female×repertoire size *F*_25 571_=1.8, *p*=0.015; mean *r*=−0.09±0.06, range −0.52 to 0.56). This analysis was greatly influenced by the inclusion of close male relatives as potential mates of each focal female, which formed major outliers with respect to *k* (figure 2). After excluding close male relatives as potential mates of each female, *k* was negatively correlated with song repertoire size within individual females (figure 3; mean *r*=−0.24±0.03, range −0.49 to 0.10); individual females were on average less closely related to males with larger song repertoire sizes.

## 4. Discussion

### (a) Song repertoire size and kinship

Since the exact membership of Mandarte's song sparrow population is known for each year and substantial pedigree data exist, this population permits unusually comprehensive investigation of natural variation in kinship among potential mates and therefore of relationships between kinship and secondary sexual ornamentation. In 2003, a male's mean kinship (*k*_m_) with the female population, both including and excluding close female relatives, was negatively correlated with its song repertoire size; repertoire size predicted 18–32% of variation in *k*_m_. A male's song repertoire size therefore indicated its mean kinship with the overall set of potential mates.

The existence of a population-level correlation between repertoire size and *k*_m_ does not necessarily imply that individual females will be consistently less closely related to males with larger repertoires. Indeed, considering all 22 recorded males as potential mates of each female, *k* was not consistently correlated with repertoire size within individual females. However, estimated within-female correlations were greatly (and in some cases overwhelmingly) influenced by the inclusion of close male relatives as potential mates of each female. Since animals are widely suggested to ‘recognize’ or otherwise avoid mating with close relatives (e.g. by pheromone comparison, call recognition or sex-biased natal dispersal; Pusey & Wolf 1996; Komdeur & Hatchwell 1999; Petrie *et al*. 1999; Mateo & Johnston 2000; Tregenza & Wedell 2000), it may be biologically inaccurate to include close relatives in the set of potential mates to be differentiated by reference to secondary sexual ornamentation. After excluding close relatives from the set of potential mates, *k* was on average negatively correlated with song repertoire size within individual females.

Overall, these data suggest that by following the dual mate choice strategy of preferring males with larger song repertoires while otherwise avoiding close relatives, female song sparrows could on average acquire relatively unrelated mates. Since the 22 recorded males did not differ from the 9 unrecorded males in *k*_m_ (means 0.079±0.008 and 0.071±0.010, respectively, *t*_29_=1.0, *p*=0.33), there is no clear expectation that correlations between repertoire size, *k*_m_ and *k* observed across the recorded males should not hold across the entire male population. Furthermore, the 22 recorded males arguably comprised the full set available for primary mate choice in 2003 (§2). These data suggest that on Mandarte, a directional female preference for males with larger song repertories, particularly when coupled with avoidance of close relatives, would on average translate into choice for relatively unrelated mates and therefore for relatively outbred offspring. Indeed, across 20 males whose songs were recorded that bred in 2003, offspring *f* was negatively correlated with paternal repertoire size (*r*_s_=−0.53, *n*=20, *p*=0.016; two recorded males remained unmated). Since major fitness components decline with increasing *f* in song sparrows and other species (Keller 1998; Crnokrak & Roff 1999; Keller & Waller 2002), such a directional mating preference is likely to translate into a non-additive genetic fitness benefit for the average female. Since there is no evidence that, on Mandarte, extra-pair paternities occur systematically with respect to relatedness or alter the degree of reproductive skew (O'Connor *et al*. 2006; Reid *et al*. 2007), these conclusions are unlikely to be biased by paternity error in the pedigree.

**Figure 4:**
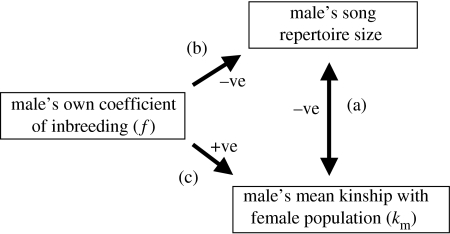
One pathway explaining the observed negative (−ve) correlation between a male's song repertoire size and mean kinship (*k*_m_) with the female population (link a). On Mandarte, a male song sparrow's song repertoire size is negatively (−ve) correlated with its own coefficient of inbreeding (*f*, link b), while *f* is positively (+ve) correlated with *k*_m_ (link c).

The possibility that directional mating preferences for more ornamented males might confer non-additive genetic benefits is not typically considered. Rather, a clear dichotomy is often drawn between female choice for additive good genes by means of directional preferences for increased ornamentation, and female choice for non-additive compatible genes by means of female-specific immunological, cytological, auditory or olfactory comparison (Widemo & Sæther 1999; Tregenza & Wedell 2000; Colegrave *et al*. 2002; Mays & Hill 2004; Neff & Pitcher 2005). This dichotomy is drawn because there is no straightforward expectation that ornamentation should indicate *k* or any other component of genetic ‘compatibility’ (Puurtinen *et al*. 2005), or that individual males should provide non-additive genetic benefits to all females (rather than specific individual females; Neff & Pitcher 2005). It is therefore instructive to consider why song repertoire size was correlated with *k*_m_ (and *k*) in song sparrows. Since repertoire size is unlikely to influence *k*_m_ directly (or vice versa), some indirect correlative pathway is presumably responsible. One possible pathway is shown in figure 4. On Mandarte, song repertoire size is negatively correlated with a male's own *f*, probably representing direct inbreeding depression in the expression of this secondary sexual trait (Reid *et al*. 2005). In addition, *k*_m_ is positively correlated with individual *f*; relatively inbred parents are on average more closely related to the set of potential mates and therefore intrinsically likely to produce relatively inbred offspring (Reid *et al*. 2006). This correlation arises as a consequence of the population's relatedness structure, where occasional immigrants interbreed with existing natives and lineages differ in fitness (Reid *et al*. 2006). One explanation for the observed negative correlation between song repertoire size and *k*_m_ is therefore that song repertoire size is negatively correlated with male *f*, while *f* is positively correlated with *k*_m_ (figure 4). Therefore, while other pathways may also exist, the observed correlation between song repertoire size and *k*_m_ can be rationalized as a consequence of inbreeding depression in ornamentation expressed in the context of the intrinsic relatedness structure of Mandarte's song sparrow population.

### (b) Generality

It is difficult to assess the generality of the correlations between ornamentation and kinship (and the consequent possibility that non-additive genetic benefits could result from directional female preferences) apparent in song sparrows since few comparable data are available. As a first step, the probable generality of the conditions underlying the pathway suggested in figure 4 can be considered. For this pathway to operate, first, inbreeding must occur such that within-population variance exists in *f*. This may be common in small, fragmented or highly structured natural populations, and in captive populations that are often used for mate choice experiments (Keller & Waller 2002; Smith *et al*. 2006).

Second, there must be inbreeding depression in the expression of the secondary sexual trait. Although surprisingly few studies have explicitly tested for such effects, particularly in free-living populations, inbreeding depression has been observed in ornamentation, courtship behaviour and male mating success (Maynard Smith 1956; Aspi 2000; Höglund *et al*. 2002; Joron & Brakefield 2003; van Oosterhout *et al*. 2003). Ornamentation can also decline with multi-locus heterozygosity and mean *d*^2^ (Foerster *et al*. 2003; Marshall *et al*. 2003). Furthermore, inbreeding may severely affect immunology, metabolism and stress response (Reid *et al*. 2003; Kristensen *et al*. 2005), and secondary sexual traits may be particularly sensitive to such components of ‘condition’ (Cotton *et al*. 2004; Tomkins *et al*. 2004). Finally, inbreeding depression is predicted to be most severe in traits under directional selection, which is likely to include secondary sexual traits (Falconer & Mackay 1996). Therefore, in populations where inbreeding occurs, inbreeding depression in ornamentation should perhaps be expected.

Third, to generate a fitness benefit of producing relatively outbred offspring, fitness must decline with inbreeding. Such inbreeding depression is widespread in natural and captive populations and is frequently severe (Crnokrak & Roff 1999; Keller & Waller 2002).

Fourth, population members must vary in their relatedness to the set of potential mates. Although few empirical data are available, such variance seems likely to be common in structured populations, where immigrants interbreed with existing natives and, of particular relevance in the context of sexual selection, in populations with high reproductive skew.

Finally, there must be intrinsic population structure such that relatively inbred individuals are more closely related to the set of potential mates. Such structure can cause parent–offspring resemblance with respect to *f* (Reid *et al*. 2006), a possibility that is not generally considered in the context of mate choice (see Mays & Hill 2004; Neff & Pitcher 2005). Although further investigation is required, such correlations may arise under a range of conditions in structured populations (Bensch *et al*. 2006; Reid *et al*. 2006). Therefore, while further empirical and theoretical studies are clearly required, it appears possible that correlations between ornamentation and kinship such as those observed in song sparrows may occur elsewhere, at least in structured populations where inbreeding occurs.

### (c) Implications for genetic models of sexual selection

The fitness costs and benefits that modulate the evolution of directional female preferences for more ornamented males are likely to be multiple and context-dependent, and to include direct and indirect effects (Andersson 1994; Jia & Greenfield 1997; Kokko *et al*. 2003). Since male song sparrows defend breeding territories and provide parental care, mate choice may substantially reflect direct benefits in this species. However, it is thought-provoking to consider the possible evolutionary implications of directional female preferences for non-additive genetic benefits. The average effect size for the within-female correlation between male repertoire size and *k* observed in song sparrows was moderate (*r*=−0.24, after excluding close relatives as potential mates), equating to an average reduction in offspring *f* of 0.003 per additional male song type (an approximately 6% reduction, given the current Mandarte average of 0.05). Given the average inbreeding depression in lifetime reproductive success (LRS) observed on Mandarte (Keller 1998), this translates into an approximately 1% average increase in offspring LRS per unit increase in paternal repertoire size. Since song sparrow repertoire sizes varied from 5 to 11 on Mandarte (Reid *et al*. 2005), a female preference for the most versus least ornamented male could therefore increase offspring fitness by approximately 6% on average. While these effects are in one sense small, they may be substantial evolutionary forces (given a low cost of mate choice) and are comparable to the postulated fitness benefit of female choice for additive good genes (given that the heritability of fitness is expected to be low; Alatalo *et al*. 1998; Møller & Alatalo 1999; Neff & Pitcher 2005). Furthermore, such non-additive genetic fitness benefits may be larger in populations where inbreeding depression is more severe than on Mandarte, where variance in relatedness is greater (e.g. where reproductive skew is great) or where ornamentation is more variable. The consequences of such non-additive genetic benefits for the evolution and maintenance of directional female preferences require explicit evaluation. The existence of intrinsic correlations between genetic dissimilarity and the expression of condition-dependent traits might conceivably provide an initial fitness benefit of a directional mating preference, driving an initial system of mate choice on which further selection could then act. Furthermore, since the identity of the least closely related (and therefore most ornamented and preferred) male lineage may be inherently frequency dependent, the existence of links between ornamentation and relatedness may bear on the maintenance of genetic variance under persistent directional female mating preferences.

### (d) Implications for interpretations of empirical data

In song sparrows, paternal ornamentation was on average negatively correlated with *k*_m_ (and consequently offspring *f*). Given inbreeding depression in fitness, offspring fitness would therefore be predicted to be positively correlated with paternal ornamentation (constituting a non-additive genetic benefit of female choice). This prediction is identical to that made in the context of female choice for additive genetic benefits (Hunt *et al*. 2004). Indeed, positive correlations between paternal ornamentation and components of offspring fitness (in the absence of direct benefits) are often interpreted as evidence for additive good genes (Kirkpatrick 1996; Møller & Alatalo 1999; Hunt *et al*. 2004). In view of the patterns evident in song sparrows, empiricists should consider whether observed correlations between paternal ornamentation and offspring fitness may partly reflect non-additive genetic benefits of mate choice. This is most probable in structured populations where inbreeding occurs (see §4*b*), which may include some key empirical studies. For example, Hasselquist *et al*. (1996) documented increased survival in extra-pair offspring of great reed warblers (*Acrocephalus arundinaceous*) with large song repertoires in a population that shows inbreeding, inbreeding depression, variance in relatedness and genetic structuring (Hansson *et al*. 2002, 2004). Petrie (1994) documented increased survival in cross-fostered offspring of peacocks with elaborate tails in a small structured peafowl (*Pavo cristatus*) population, where relatives coexist and inbreeding is probable (Petrie *et al*. 1999). These studies are frequently cited, either explicitly or implicitly, as key empirical support for additive good genes models of female choice (e.g. Jones *et al*. 1998; Krokene *et al*. 1998; Møller & Alatalo 1999). The correlations between ornamentation and kinship observed in song sparrows suggest that more circumspect interpretation may be required, at least until the possibility of non-additive genetic effects is further investigated.

### (e) Conclusion

It is commonly assumed that non-additive components of genetic quality cannot be intrinsically correlated across fathers and offspring, and therefore cannot be obtained via unanimous directional female preferences for more ornamented males (Mays & Hill 2004; Neff & Pitcher 2005; Puurtinen *et al*. 2005). The correlations between song repertoire size, *k*, paternal *f* and offspring *f* observed in song sparrows (see also Reid *et al*. 2005, 2006) suggest that these assumptions may be simplistic in the context of structured populations (see also Bensch *et al*. 2006). Kokko & Brooks (2003) noted that sexual selection may impact population structure and dynamics. Current data indicate that population structure may itself influence the genetic benefits of sexual selection, and should therefore be explicitly incorporated into models investigating the evolution and maintenance of directional mating preferences and elaborate ornamentation.
